# Barriers and enablers to exercise adherence in people with nonspecific chronic low back pain: a systematic review of qualitative evidence

**DOI:** 10.1097/j.pain.0000000000003234

**Published:** 2024-04-16

**Authors:** Yannick L. Gilanyi, Brishna Shah, Aidan G. Cashin, Mitchell T. Gibbs, Jessica Bellamy, Richard Day, James H. McAuley, Matthew D. Jones

**Affiliations:** a School of Health Sciences, Faculty of Medicine and Health, University of New South Wales, Sydney, Australia; b Centre for Pain IMPACT, Neuroscience Research Australia, Sydney, Australia; c St Vincent's Clinical Campus, Faculty of Medicine and Health, University of New South Wales, Sydney, Australia

**Keywords:** Systematic review, Qualitative, Low back pain, Exercise

## Abstract

Exercise is a first-line treatment for chronic low back pain (CLBP), reducing pain and disability in the short term. However, exercise benefits decrease over time, with a lack of long-term exercise adherence a potential reason for this. This study aimed to synthesize the perceptions and beliefs of individuals with CLBP and identify their barriers and enablers to exercise adherence. We searched CENTRAL, Embase, CINAHL, SPORTDiscus, PubMed, PsycINFO, and Scopus databases from inception to February 28, 2023, for qualitative studies that explored the factors influencing exercise adherence for people with CLBP. A hybrid approach combining thematic synthesis with the Theoretical Domains Framework was used to analyze data. We assessed methodological quality using the Critical Appraisal Skills Programme checklist and the level of confidence of the themes generated using the Confidence in the Evidence from Reviews of Qualitative Studies. Twenty-three papers (n = 21 studies) were included (n = 677 participants). Four main themes affected exercise adherence: (1) exercise, pain, and the body, (2) psychological factors, (3) social factors, and (4) external factors. These themes contained 16 subthemes that were predominantly both barriers and enablers to exercise adherence. The individual's experiences of barriers and enablers were most appropriately represented across a spectrum, where influencing factors could be a barrier or enabler to exercise adherence, and these could be specific to pre-exercise, during-exercise, and post-exercise situations. These findings may be used to improve exercise adherence and ultimately treatment outcomes in people with CLBP.

## Abstract 2

Supplemental Digital Content is Available in the Text.

## 1. Background

Low back pain is the leading cause of global disability,^25^ which poses a major economic burden that affects both healthcare systems and gross domestic product.^53,63,76^ Most of the costs of low back pain are incurred by people with chronic low back pain (CLBP; pain lasting more than 3 months' duration). The most commonly recommended treatment for CLBP is exercise because it reduces pain and functional disability in the short term.^31,32^ However, at intermediate- and long-term follow-ups, the benefits of exercise on pain and functional disability are no longer clinically meaningful.^31^

Adherence to exercise may be an important factor influencing the diminishing benefits of exercise for CLBP over time. For example, single sessions of exercise do not reliably decrease pain,^57,65^ implying that a greater amount of sustained exercise could be important. However, higher levels of exercise adherence result in faster and more significant improvements in pain and function^34,49^ and are important for sustaining improvements in CLBP.^26^ Despite this, up to 70% of people with CLBP do not adhere to prescribed home exercises^8^ and are also significantly less likely to be physically active compared with those without CLBP.^78^ To improve the long-term efficacy of exercise treatment, it is important to gain a better understanding of the factors that influence exercise adherence in people with CLBP.

Previous research in people with chronic health conditions has identified over 200 factors that are associated with the behaviour of exercise adherence.^70^ This has led to the development of several health psychology frameworks to try and understand the process of adherence.^7,66^ A common concept across these frameworks is that barriers and enablers perceived by people influence their adherence to exercise.^6,60^ Barriers and enablers are defined as influential factors that either discourage/hinder people from completing exercise (barrier) or encourage/facilitate them in completing exercise (enabler). Although many barriers and enablers could affect exercise adherence,^17^ it is important to understand the barriers and enablers that are unique to people with CLBP. For example, a fear that exercise may worsen pain may be a greater barrier for people with CLBP compared with other conditions where pain is not an important symptom.^11^

Previous reviews^8,35,50^ have investigated the effects of various interventions on exercise adherence in chronic musculoskeletal pain populations, with one review specifically examining barriers and enablers to exercise in those with CLBP.^69^ However, many studies included in this review emphasised the opinions of healthcare providers rather than the perspectives of individuals with CLBP,^69^ reflecting poor alignment with key elements of pain management such as shared decision making and person-centred care.^44^

This systematic review aimed to identify the barriers and enablers to exercise adherence from the perspective of individuals with CLBP. It is hoped that findings from this study will be used to refine exercise interventions for people with CLBP to improve adherence.

### 1.1. Objectives

To identify barriers and enablers to exercise adherence for people with CLBP.

## 2. Methods

This study is a systematic review of qualitative studies. We prospectively registered the protocol for this systematic review on the Open Science Framework (https://osf.io/2prwz/). A critical realist paradigm was taken, which posits that an objective reality exists that can be changed and manipulated (eg, the behaviour of exercise adherence can be changed); however, our knowledge of this reality cannot be absolute as it is bound by our social, cultural, and historical contexts (ie, the results from this review will not represent a universal knowledge).^12,61^ Alongside this, a thematic synthesis approach was used to identify and synthesize perspectives of individuals with CLBP. We have reported our findings in accordance with the “Enhancing transparency in reporting the synthesis of qualitative research” (ENTREQ) guidelines.^74^

### 2.1. Search strategy

We searched the following databases from inception to February 28, 2023: CENTRAL, Embase, CINAHL, SPORTDiscus, PubMed, PsycINFO, and Scopus. We extended searches by conducting forward and backward citation tracking of included studies and relevant systematic reviews. The full search strategy can be found in Supplementary Data 1 (available at http://links.lww.com/PAIN/C34).

### 2.2. Eligibility criteria

#### 2.2.1. Types of studies

We included primary studies that examined the experiences of exercise adherence in people with CLBP using both qualitative methods for data collection (eg, focus groups, individual interviews, and open-ended survey questions) and data analysis (eg, thematic analysis and framework analysis). We included mixed-methods studies where it was possible to extract the qualitative data alone. We excluded studies that did not report the experiences of the people exercising (eg, studies only of health professionals' perspectives) and studies that were not written in English.

#### 2.2.2. Participants

We included studies of adults (older than 18 years) with CLBP in any country or health setting. Chronic low back pain was defined as pain between the 12th rib and gluteal fold, persisting for greater than 3 months.^27^ We excluded studies where greater than 20% of participants met the definition of spine-related leg pain with radiculopathy,^40^ where the average duration of low back pain was <3 months, or where CLBP was attributable to a specific pathology, such as infection, neoplasm, metastasis, inflammatory disease, or fractures.

#### 2.2.3. Interventions

We included studies that investigated general exercise practice/habits of participants and studies that prescribed an exercise intervention/program. Exercise was defined as “a specific type of physical activity that is planned, structured, and repeatedly done to improve or maintain physical fitness.”^15^ Exercise adherence was defined as the behaviour of consistently maintaining an exercise plan or goal.^14^ Subsequently, we inferred that all intentional exercise (single sessions, short- and long-term programs) was part of the overall behaviour of exercise adherence, as each session was intentionally meeting part of the goal. Accordingly, participants' experiences of some short-term exercise interventions were extrapolated to represent barriers and enablers to longer-term exercise adherence. Therefore, all exercise experiences were included in the review with no limitations placed on the exercise or data collection time frames. Studies investigating physical activity that did not distinguish between exercise and activities of daily living were excluded because barriers and enablers for intentional physical activity/exercise are likely to be different to unintentional or incidental activities of daily living.^16^

### 2.3. Study selection

We uploaded search results to Covidence.^1^ Two authors (Y.L.G. and B.S.) independently screened the title and abstracts of each study to determine eligibility. The same authors assessed the eligibility of full-text studies, recording reasons for exclusion. Disagreements were resolved through discussion and arbitration of a third author when necessary (M.D.J.).

### 2.4. Data extraction

Two authors (Y.L.G. and B.S.) independently extracted data from each study into a customised Microsoft Excel spreadsheet. Descriptive data were extracted for the following: (1) study characteristics (country, setting, study design, qualitative framework, objectives, data collection methods, and sample size); (2) participant details (age, sex, duration of CLBP, education, cultural background, socioeconomic status, occupational status, and comorbidities); and (3) intervention details (type(s) of exercise completed, frequency of exercise, and exercise intensity). Qualitative data extraction included participant's quotes from within the results or discussion sections of the included studies. Authors' explanations of quotes were also extracted when necessary to provide contextual information.

### 2.5. Data synthesis and analysis

We used a hybrid approach^10^ combining a 3-stage process of thematic synthesis as described by Thomas and Harden^73^ with a deductive approach using the Theoretical Domains Framework (TDF) of behaviour change.^5^ This hybrid approach was used to provide an accurate representation of the data through the inductive analysis whilst simultaneously presenting the data through a theory-driven lens.^10^ The TDF was chosen because it provides a comprehensive theory-informed approach to identify factors associated with behaviour change, which can be targeted by interventions seeking to change these behaviours.^55^ The first stage of thematic synthesis comprised an inductive process whereby 2 authors (Y.L.G. and B.S.) independently performed line-by-line coding of all qualitative data. In the second stage, the same 2 authors discussed their codes, finding similarities to create descriptive themes that reflected findings and patterns represented across the included studies. In the final stage, we developed analytical themes by going beyond the content of the original included articles and synthesized descriptive themes into major themes and developed subthemes as barriers and enablers that reflected the aim of the study. One theme identified in this process was that barriers and enablers were often time-dependent. Therefore, as part of the deductive analysis, subthemes were mapped onto time points of pre-exercise, during-exercise, and post-exercise. This was conducted through analysing the context, language, and tenses used by the people reporting their barriers and enablers. Subthemes (barriers and enablers) were mapped onto relevant domains of the TDF to provide a theory-informed guide for future implementation of exercise adherence behaviour change for people with CLBP. Through all stages of analysis, consensus meetings were held between authors Y.L.G., B.S., M.D.J. and J.H.M.

Two review authors (Y.L.G. and M.D.J.) independently assessed methodological limitations for each study using the Critical Appraisal Skills Programme Qualitative Studies checklist.^2^ We assessed whether primary articles reported clear information on the following items: study aims, methodology, design, recruitment strategy, data collection, data analysis, reflexivity, ethical considerations, findings, and value of research. Each item was rated as “yes,” “can't tell,” or “no,” with comments written to inform the severity or justification of the missing items. Studies were then rated as having “no or very minor concerns,” “minor concerns,” “moderate concerns,” or “serious concerns” regarding methodological limitations. Authors used their qualitative research and content expertise to judge both the number of items that studies failed to report and the subsequent impact that this may have on the quality of evidence.^42,48^ Disagreements were resolved through discussion with a third author.

The Grading of Recommendations Assessment, Development, and Evaluation Confidence in the Evidence from Reviews of Qualitative Research (GRADE-CERQual)^41^ approach was used to assess confidence in the themes generated. Two authors (Y.L.G. and M.D.J.) independently assessed the level of confidence as the extent to which themes were a reasonable representation of the phenomenon of interest. For each theme, the following domains were judged as having “no or very minor limitations,” “minor limitations,” “moderate limitations,” or “serious limitations”: methodological limitations (concerns about the design or conduct of the primary studies), relevance (applicability of findings from the primary studies to the review question), coherence (how clearly the findings from the primary studies match those from the review), and adequacy (richness and quantity of data supporting the review finding). Each domain was judged following recommendations provided by the GRADE-CERQual guidelines.^18,29,48,52^ A consensus meeting was held to discuss the overall level of confidence in each theme generated as “high,” “moderate,” “low,” or “very low.”

### 2.6. Review author reflexivity

The review team is composed of researchers and clinicians with experience in exercise physiology, psychology, and medicine, with all authors having experience in chronic pain research. Authors Y.L.G., M.D.J., M.T.G., J.B., and J.H.M. had experience conducting qualitative research. The authors maintained a reflexive stance throughout all stages of the review process by regularly discussing and critically analyzing decisions as well as examining how their own biases were influencing the findings.

## 3. Results

**Figure 1.**
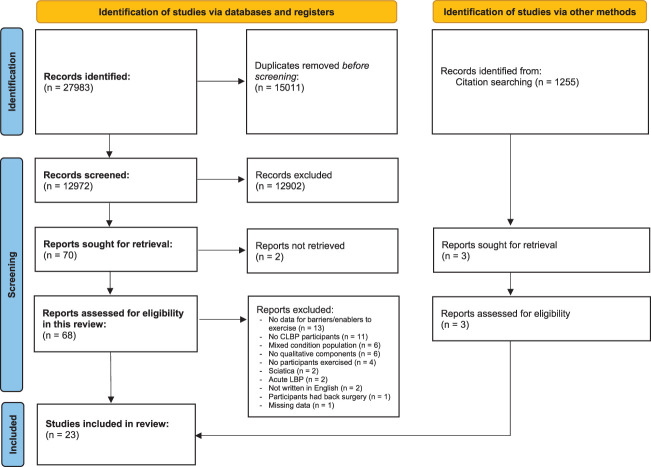
Prisma 2020 flow diagram outlining the study identification, screening, and inclusion processes. CLBP, chronic low back pain.

The study screening and selection process is outlined in Figure 1. Overall, 27,983 records were identified and 15,011 were removed before 12,972 studies were screened at title and abstract level. We screened 70 full texts for eligibility and included 23 papers (from 21 studies) (Fig. 1).

Included studies reported data from 677 participants aged 18 to 97 years. Qualitative data collection methods included individual interviews (n = 16), focus groups (n = 3), individual interviews and focus groups (n = 1), and written responses to open-ended questions (n = 1). A range of exercise experiences were collected, including from participants involved in observational or randomised controlled trial interventions (n = 9), programs prescribed by a professional in the community (n = 8), people with exercise experience (n = 3), and a mix of people with some or no exercise experience (n = 1). The characteristics of each study are presented in Table 1.

**Table 1:** Study characteristics.

Study ID (country)	Sample size	Participant characteristics	Data collection	Exercise experiences
Angel et al., 2012^4^ (Denmark)	20	Age (mean) = 46.9 ± 10.5 y Sex = 13 W/7 M CLBP duration = N/A	Recruited from = rheumatologist, nested in RCT Method = individual interviews	Intervention = exercise plan created with an occupational physician
Boutevillain et al., 2017^11^ (France)	29	Age = N/A Sex = 10 W/19 M CLBP duration = <5 y = 13 people 6-10 y = 8 people >10 y = 8 people	Recruited from = hospital Method = focus groups and individual interviews	Community = mixed exercise experiences
Buijs et al., 2009^13^ (the Netherlands)	20	Age (mean) = 46 y Sex = 11 W/9 M CLBP duration = N/A	Recruited from = hospital, nested in RCT Method = individual interviews	Intervention = up to 12 wk of 2× 1-h group exercise
Cook and Hassenkamp 2000^19^ (the United Kingdom)	7	Age (mean) = 42.3 y Sex = 4 W/3 M CLBP duration = 8.6 y	Recruited from = hospital Method = individual interviews	Community = 2× per week for 4 wk, back rehabilitation group
Cooper et al., 2008^20,21^^,^* (Scotland)	25	Age (range) = 18-34 y = 3 people 35-50 y = 8 people 51-65 y = 14 people Sex = 20 W/5 M CLBP duration = N/A	Recruited from = hospital Method = individual interviews	Community = exercise from physiotherapist or self-taught
Crowe et al., 2010^22^ (New Zealand)	64	Age (mean) = 55.1 Sex = 31 W/33 M CLBP duration = 25-80 y	Recruited from = community newsletter and physiotherapy clinics Method = individual interviews	Community = exercise from physiotherapist or learnt from others with CLBP
Dean et al., 2005^23^ (the United Kingdom)	9	Age (mean) = 39.5 Sex = N/A CLBP duration = 1-20 y	Recruited from = hospital Method = individual interviews	Community = exercise from physiotherapist
Hay et al., 2020^30^ (Canada)	10	Age (range) = 66-97 Sex = 7 W/3 M CLBP duration = 10-70 y	Recruited from = community Method = individual interviews	Community = >150 min/wk of varying types of exercise
Joyce et al., 2022a^36^ ( the United States)	12	Age (mean) = 51.9 ± 8.6 Sex = 11 F/1 M CLBP duration = N/A	Recruited from = medical centre, nested in RCT Method = individual interviews	Intervention = 60 min/wk of aerobic exercise and stabilisation or flexion/extension exercise for 12 wk
Joyce et al., 2022b^37^ ( the United States)	18	Age (mean) = 49.6 Sex = 12 W/6 M CLBP duration = N/A	Recruited from = medical centre, nested in RCT Method = individual interviews	Intervention = 75 min/wk of yoga for 12 wk
Keen et al., 1999^39^ (the United Kingdom)	27	Age (range) = 18-29 Sex = 17 W/10 M CLBP duration = <1 y = 3 people 1-5 y = 9 people 6-10 y = 6 people 11-15 y = 5 people >16 y = 4 people	Recruited from = general practitioner practice, nested in RCT Method = individual interviews	Intervention = 2× 60 min/wk progressive exercise program for 4 wk (strength, flexibility and aerobic)
Liddle et al., 2007^43^ (Northern Ireland)	18	Age (range) = 18-40 y = 6 people 41-55 y = 9 people 55-65 y = 3 people Sex = 14 W/4 M CLBP duration = N/A	Recruited from = university advertisement Method = focus groups	Community = had received exercise treatment and advice from a health professional
Mathy et al., 2015^45^ (Belgium)	30	Age (mean) = 42 ± 11.5 Sex = 16 W/14 M CLBP duration = 10.5 ± 10.7 y	Recruited from = hospital Method = individual interviews	Community = action phase† of exercising
Morris et al., 2004^47^ (the United Kingdom)	6	Age (mean) = 52 Sex = 3 W/3 M CLBP duration = 38-61 y	Recruited from = hospital Method = individual interviews	Intervention = 4× 2-h exercise sessions
Palazzo et al., 2016^54^ (France)	29	Age (mean) = 54 Sex = 17 W/12 M CLBP duration = 4.9 ± 3.8 y	Recruited from = hospital Method = individual interviews	Community = prescribed home exercise program for at least 8 wk
Riipinen et al., 2022^59^ (Wales)	10	Age (mean) = 48 Sex = 4 W/6 M CLBP duration = 7.2 y	Recruited from = chiropractic practice Method = individual interviews	Community = current or previous experience of exercising
Saner et al., 2018^62^ (the Netherlands)	44	Age (mean) = 44.2 ± 13.1 Sex = 19 W/25 M CLBP duration = 9 y	Recruited from = hospital/private practice, nested in RCT Method = 3 open-ended survey questions	Intervention = 2× 30 min/wk movement control and resistance training for 12 wk, then home program for the following year
Slade et al., 2009^67,68^^,^* (Australia)	18	Age (mean) = 51.2 ± 10 Sex = 12 W/6 M CLBP duration = 20.3 ± 13 y	Recruited from = community Method = focus groups	Community = participated in an exercise program
Sokunbi et al., 2010^71^ (the United Kingdom)	9	Age (mean) = 42.6 ± 8.8 Sex = 6 W/3 M CLBP duration = N/A	Recruited from = N/A, nested in RCT Method = focus groups	Intervention = 1, 2 or 3× per week spinal stabilisation program for 6 wk
Stilwell et al., 2017^72^ (Canada)	6	Age (mean) = 34.5 ± 14.4 Sex = 3 W/3 M CLBP duration = 10 ± 8.3 y	Recruited from = chiropractic practice Method = individual interviews	Community = received exercise instruction or advice
Yardley et al., 2010^77^ (the United Kingdom)	24	Age (mean) = 46.3 ± 9.4 Sex = 13 W/11 M CLBP duration = N/A	Recruited from = general practitioner practice, nested in RCT Method = individual interviews	Intervention = Alexander technique and/or exercise prescription from a doctor

* Studies that included 2 papers.

† Action phase of Health Action Process Approach model.^64^

CLBP, chronic low back pain; RCT, randomized controlled trial.

### 3.1. Assessment of methodological limitations

The assessment of the methodological limitations in each study^2^ can be found in Table 2. From the 23 papers included, 12 had no or very minor methodological concerns, 7 had minor concerns, 3 had moderate concerns, and 1 had serious concerns. Author reflexivity was poorly reported, with 18 of 23 papers providing no description of researcher-participant relationships. In addition, 14 of 23 studies did not provide information justifying the research design used in their studies. Reporting and conduct of methods, sampling, data collection, ethics, data analysis, and findings sections had very few methodological limitations across the included studies.

**Table 2:** Methodological limitations of each study.

Study	Aims	Qualitative methodology	Research design	Sampling	Data collection	Reflexivity	Ethics	Data analysis	Findings	Value of research	Assessment
Angel et al. (2012)^4^	Y	Y	Y	Y	Y	?	Y	Y	Y	Y	No/very minor
Boutevillain et al. (2017)^11^	Y	Y	Y	Y	Y	?	Y	Y	Y	Y	No/very minor
Buijs et al. (2009)^13^	Y	Y	Y	Y	Y	?	Y	Y	Y	Y	No/very minor
Cook and Hassenkamp (2000)^19^	Y	Y	Y	Y	Y	?	Y	Y	Y	Y	No/very minor
Cooper et al. (2008)^20^	Y	Y	?	Y	Y	Y	Y	Y	Y	Y	No/very minor
Cooper et al. (2009)^21^	Y	Y	?	Y	Y	Y	Y	Y	Y	Y	No/very minor
Crowe et al. (2010)^22^	Y	Y	?	Y	Y	?	Y	Y	Y	Y	Minor
Dean et al. (2005)^23^	Y	Y	Y	Y	Y	?	Y	Y	Y	Y	No/very minor
Hay et al. (2020)^30^	Y	Y	Y	Y	Y	Y	Y	Y	Y	Y	No/very minor
Joyce et al. (2022a)^36^	Y	Y	Y	Y	Y	?	Y	Y	Y	Y	No/very minor
Joyce et al. (2022b)^37^	Y	Y	?	N	Y	?	Y	Y	Y	Y	Moderate
Keen et al. (1999)^39^	Y	Y	?	Y	?	?	Y	Y	Y	Y	Moderate
Liddle et al. (2007)^43^	Y	Y	?	Y	Y	?	Y	Y	Y	Y	Minor
Mathy et al. (2015)^45^	Y	Y	?	Y	Y	?	Y	Y	Y	Y	Minor
Morris et al. (2004)^47^	Y	Y	?	Y	Y	Y	Y	Y	Y	Y	No/very minor
Palazzo et al. (2016)^54^	Y	Y	?	Y	Y	?	Y	Y	Y	Y	Minor
Riipinen et al. (2022)^59^	Y	Y	Y	Y	Y	?	Y	Y	Y	Y	No/very minor
Saner et al. (2018)^62^	Y	?	?	Y	?	?	Y	Y	Y	Y	Serious
Slade et al. (2009a)^67^	Y	Y	?	Y	Y	?	Y	Y	Y	Y	Minor
Slade et al. (2009b)^68^	Y	Y	?	Y	Y	?	Y	Y	Y	Y	Minor
Sokunbi et al. (2010)^71^	Y	Y	?	Y	?	?	Y	Y	Y	Y	Moderate
Stilwell et al. (2017)^72^	Y	Y	Y	Y	Y	Y	Y	Y	Y	Y	No/very minor
Yardley et al. (2010)^77^	Y	Y	?	Y	Y	?	Y	Y	Y	Y	Minor

Y = The item was included in study, ? = Can't tell if the item was included in the study, N = The item was not included in the study.

### 3.2. Themes

**Figure 2.**
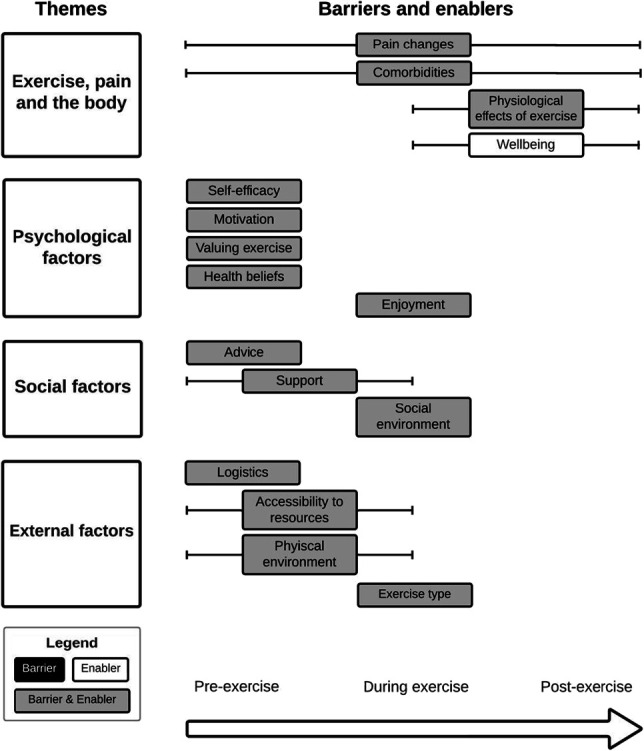
Barries and enablers to exercise adherence at different time points. The horizontal bars represent the exercise time points (pre, during, and/or post-exercise) that barriers and enablers span across.

Four themes and 16 subthemes were identified (Fig. 2). Below, the data from each theme are discussed in relation to barriers and enablers to exercise adherence. Barriers and enablers within each theme are also categorised into time points relative to exercise completion: pre-exercise, during-exercise, and post-exercise. Relevant quotes for each barrier and enabler at their relevant time points can be found in Supplementary Data 2 (available at http://links.lww.com/PAIN/C34).

#### 3.2.1. Theme 1: exercise, pain, and the body

Data in this theme captured the results from studies on how the interconnected experiences of exercise, pain, and the body affected exercise adherence. Barriers and enablers included pain changes, other comorbidities, the physiological effects of exercise, and improved well-being.

##### 3.2.1.1. Pain changes

Pain (and/or discomfort) hindered exercise adherence in the pre-exercise and during-exercise stages, with studies reporting individuals would not exercise due to their pain or would cease exercise if they experienced an increase in their pain intensity. Pain changes continued to be a barrier post-exercise where participants who did not receive sufficiently meaningful reductions in their pain intensity from exercise did not deem it worthwhile and thus stopped adhering. Conversely, experiencing meaningful pain relief both during and after exercise was reported as an enabler to adherence.

##### 3.2.1.2. Other comorbidities

The presence and impacts of other comorbidities (eg, arthritis and depression) were barriers to adherence before and during exercise, whereas any improvements in comorbidities (eg, weight loss) as a result of exercise were viewed as an enabler to adherence.

##### 3.2.1.3. Physiological effects of exercise

The physiological responses experienced because of exercise, for example, sweating, increased heart rate, and fatigue, were reported as unpleasant and barriers both during and shortly after exercise. However, the physiological effects experienced after sustained exercise such as improvements in fitness (aerobic capacity) and strength were reported as enablers to adherence because of the resulting improvements in functional capacity and exercise ability.

##### 3.2.1.4. Improved well-being

Improvements in feelings of well-being during and after exercise were viewed as enablers to adherence. Well-being was the only unidimensional subtheme, reported only as an enabler.

#### 3.2.2. Theme 2: psychological factors

Data in this theme captured the results from studies on how an individual's psychological state and intrapersonal processes affected their exercise adherence. Barriers and enablers included levels of self-efficacy and motivation, value placed on exercise, health beliefs, and enjoyment.

##### 3.2.2.1. Self-efficacy

Higher levels of self-efficacy in the pre-exercise stage enabled individuals to have the confidence to engage in exercise and subsequently adhere. Conversely, lower levels of self-efficacy pre-exercise were a barrier to adherence as individuals did not have the confidence to exercise safely or without exacerbating pain. Studies most commonly reported that individuals had or lacked the confidence to complete specific exercise movements correctly or safely despite their pain. The presence of a health professional along with helpful instructions or feedback on how to exercise were the most commonly described positive influencers of self-efficacy.

##### 3.2.2.2. Motivation

Higher and lower levels of motivation in the pre-exercise stage were associated with an increased or decreased desire to exercise, respectively, and thus influenced exercise adherence. This cyclical nature was present in many studies where motivation could be the cause or result of barriers and enablers to exercise adherence. Thus, it was presented intertwined with other barriers and enablers that could be extrinsic rewards or consequences of exercise adherence. However, studies also reported that motivation was a separate barrier and enabler, an intrinsic driving factor to exercise that people possessed or lacked.

##### 3.2.2.3. Value placed on exercise

The value placed on exercising over other actions influenced decision making in the pre-exercise stage. Individuals who placed high value on exercise overcame barriers and exercised because of its perceived importance. By contrast, individuals who did not value exercise, despite experiencing other enabling factors, did not adhere to their exercise treatment.

##### 3.2.2.4. Health beliefs

Beliefs regarding the effects of exercise on one's overall health and specifically whether it may impact the pathology of their CLBP, were key barriers or enablers to exercise adherence in the pre-exercise stage. Overall, beliefs that exercise would improve function and health, and decrease pain were enablers to exercise adherence, whereas beliefs that exercise could cause further damage and exacerbate pain was a barrier to adherence.

##### 3.2.2.5. Enjoyment

Finally, enjoyment was an enabler to adherence during exercise, whereas a lack of enjoyment was a barrier.

#### 3.2.3. Theme 3: social factors

Data in this theme captured the results from studies on how an individual's interpersonal interactions affected their exercise adherence. Barriers and enablers included advice and support received and the social environment.

##### 3.2.3.1. Advice

Studies reported that individuals received a variety of advice in the pre-exercise stage that was either a barrier or enabler to exercise adherence. Enabling advice consisted of healthcare professionals recommending/prescribing exercise and clearly explaining pain education concepts as well as family members, friends, or peers (with CLBP) providing suggestions/anecdotes on the benefits of exercise for CLBP. Advice that was a barrier consisted of healthcare professionals recommending against exercising or individuals being told by health professionals or family and friends that exercise would damage their back.

##### 3.2.3.2. Support

The level of support that an individual felt they received from health professionals, family members, and friends before and during exercise was also a barrier or enabler. Individuals felt supported through verbal encouragement, having others exercise with them, being allowed autonomy, and individualised treatment, which then led to exercise adherence. However, a lack of support from family or friends and little supervision from health professionals were barriers to adherence.

##### 3.2.3.3. Social environment

The social environment experienced during exercise could be a barrier or enabler. Commonly reported attributes of a positive social environment included good rapport with the health professional, exercising with friends, family, or alone (if preferred), and a sense of community or belonging. Negative social environments that decreased adherence consisted of a lack of understanding between the health professional and individual or exercising alone or in a group where the individual felt uncomfortable.

#### 3.2.4. Theme 4: external factors

Data in this theme captured the results from studies on how external factors affected exercise adherence. Barriers and enablers included logistical factors, accessibility to resources, physical environment, and exercise type.

##### 3.2.4.1. Logistical factors

Studies reported that managing logistics and balancing a variety of responsibilities such as work, family, and other lifestyle choices was a barrier to adherence in the pre-exercise stage, whereas having free time was an enabler.

##### 3.2.4.2. Accessibility to resources

Having resources such as exercise equipment/facilities, exercise programs, and technology to facilitate exercise, and having accessibility to these resources through geographical location, transport, and finances were enablers to exercise adherence in both the pre-exercise and during-exercise stages. The absence of these resources and/or accessibility to them was a barrier to adherence.

##### 3.2.4.3. Physical environment

The physical environment was both a barrier and enabler to adherence as it affected how individuals perceived the exercise experience would be pre-exercise or how the experience was during exercise. Barriers included a lack of daylight, poor weather, and exercising inside, whereas enablers included sufficient daylight, nice weather, and exercising inside.

##### 3.2.4.4. Exercise type

The exercise type was also a barrier and enabler to exercise adherence as it affected the experience during exercise. These were dependent on individual preference. Barriers included exercise types that were difficult, unenjoyable, or boring (eg, running and types that made people feel uncomfortable or did not fit with their social identity such as weightlifting or yoga). Enablers included new and interesting exercise types, simple exercises that were not overly complex, and exercises that were fun.

### 3.3. Theoretical domains framework

**Figure 3.**
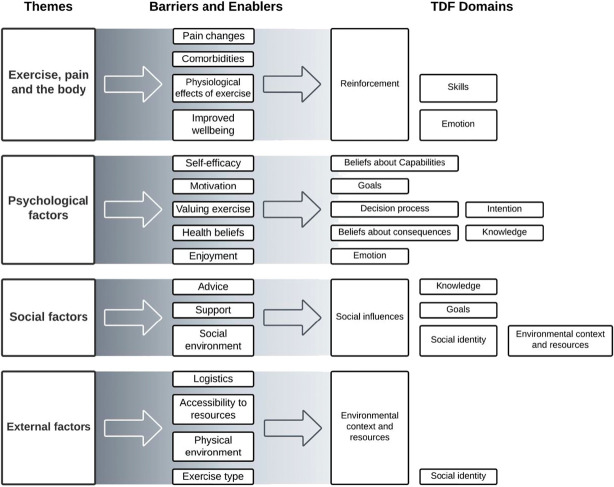
Barriers and enablers mapped to the relevant domains of the Theoretical Domains Framework (TDF).

Barriers and enablers were mapped onto 12 relevant domains of the TDF (Fig. 3). Reinforcement was a relevant domain to all the barriers and enablers within the theme “exercise, pain, and the body.” Reinforcement was defined as the process of increasing/decreasing the likelihood of exercise adherence through establishing a response to the behaviour, for example, pain relief because of exercise reinforces exercise adherence behaviour. Barriers and enablers of “psychological factors” had no common TDF domain. Instead, they often encompassed a domain themselves, for example, self-efficacy was mapped onto “beliefs about capabilities,” which was defined as the process of confidence in one's ability. Unsurprisingly, “social influences” and “environment, context, and resources” were relevant domains to all barriers and enablers within their respective themes of “social factors” and “external factors.” Other less common TDF domains linked to barriers and enablers were skills; beliefs about capabilities; goals; intention; decision processes; knowledge; beliefs about consequences; emotion; and social identity.

### 3.4. Certainty of evidence

The confidence in each theme generated according to the GRADE-CERQual assessment can be found in Table 3. Our analysis identified 4 main themes, which were each separated into barriers and enablers, resulting in 8 findings (barriers: negative physical sensations, psychological barriers, social barriers, and external barriers; enablers: exercise benefits, psychological enablers, social enablers, and external enablers). Overall, 6 findings were rated as high confidence and 2 findings were rated as moderate confidence. A detailed assessment of judgements for each component can be found in Supplementary Data 3 (available at http://links.lww.com/PAIN/C34).

**Table 3:** Summary of qualitative findings.

Summarised view finding	References	Assessment of confidence	Explanation of assessment
Enablers			
Experiencing positive bodily experiences and sensations because of exercise reinforces exercise adherence behaviour. These experiences include immediate and future pain relief, improvements in functional capacity, mood, and outcomes for other health conditions as well as exercise-specific outcomes such as strength and fitness	Angel et al.^4^ Boutevillain et al.^11^ Buijs et al.^13^ Cook and Hassenkamp^19^ Cooper et al.^20^ Cooper et al.^21^ Crowe et al.^22^ Hay et al.^30^ Joyce et al.^37^ Joyce et al.^36^ Liddle et al.^43^ Mathy et al.^45^ Morris et al.^47^ Riipinen et al.^59^ Saner et al.^62^ Slade et al.^67^ Slade et al.^68^ Sokunbi et al.^71^ Stilwell et al.^72^ Yardley et al.^77^	Moderate confidence	Minor concerns regarding methodological limitations, minor concerns regarding coherence, no/very minor concerns regarding adequacy, and minor concerns regarding relevance
Being in a psychological state or having cognitive processes/emotions that lead to exercise adherence. These include self-efficacy, motivation, enjoyment, valuing and prioritising exercise, and beliefs that exercise has a positive impact on pain outcomes	Boutevillain et al.^11^ Cooper et al.^20^ Cooper et al.^21^ Hay et al.^30^ Joyce et al.^37^ Joyce et al.^36^ Keen et al.^39^ Liddle et al.^43^ Mathy et al.^45^ Morris et al.^47^ Palazzo et al.^54^ Riipinen et al.^59^ Saner et al.^62^ Slade et al.^67^ Slade et al.^68^ Sokunbi et al.^71^ Stilwell et al.^72^ Yardley et al.^77^	High confidence	Minor concerns regarding methodological limitations, minor concerns regarding coherence, no/very minor concerns regarding adequacy, and no/very minor concerns regarding relevance
Positive social experiences with health professionals, family, friends, and others with pain support exercise adherence. These experiences include clear communication and advice around pain, support, encouragement, and a preferred social environment	Angel et al.^4^ Buijs et al.^13^ Boutevillain et al.^11^ Cook and Hassenkamp^19^ Cooper et al.^20^ Cooper et al.^21^ Crowe et al.^22^ Dean et al.^23^ Hay et al.^30^ Joyce et al.^37^ Joyce et al.^36^ Keen et al.^39^ Mathy et al.^45^ Palazzo et al.^54^ Riipinen et al.^59^ Saner et al.^62^ Slade et al.^67^ Slade et al.^68^ Sokunbi et al.^71^ Stilwell et al.^72^ Yardley et al.^77^	High confidence	Minor concerns regarding methodological limitations, no/very minor concerns regarding coherence, no/very minor concerns regarding adequacy, and minor concerns regarding relevance
External factors outside of an individual that aid the process of organizing exercise and improve the experience of exercise that reinforce exercise adherence behaviour. These include timing/logistics, accessibility to resources, and exercise environment and type	Boutevillain et al.^11^ Cooper et al.^20^ Cooper et al.^21^ Crowe et al.^22^ Hay et al.^30^ Mathy et al.^45^ Palazzo et al.^54^ Saner et al.^62^ Slade et al.^67^ Slade et al.^68^ Sokunbi et al.^71^ Yardley et al.^77^	Moderate confidence	Minor concerns regarding methodological limitations, minor concerns regarding coherence, minor concerns regarding adequacy, and no/very minor concerns regarding relevance
Barriers			
Experiencing negative bodily experiences and sensations because of exercise reinforces exercise nonadherence. These experiences include pain flare-ups and discomfort, inadequate pain relief, symptoms from other comorbidities, and the physiological reactions to exercise such as increased heart rate, heavy breathing, sweating, and fatigue	Boutevillain et al.^11^ Cook and Hassenkamp^19^ Cooper et al.^21^ Crowe et al.^22^ Hay et al.^30^ Joyce et al.^37^ Joyce et al.^36^ Liddle et al.^43^ Mathy et al.^45^ Morris et al.^47^ Palazzo et al.^54^ Riipinen et al.^59^ Saner et al.^62^ Slade et al.^67^ Slade et al.^68^ Sokunbi et al.^71^ Stilwell et al.^72^ Yardley et al.^77^	High confidence	No/very minor concerns regarding methodological limitations, no/very minor concerns regarding coherence, no/very minor concerns regarding adequacy, and no/very minor concerns regarding relevance
Being in a psychological state or having cognitive processes/emotions that hinder preparation and/or worsen the experience of exercise thus reinforcing exercise nonadherence. These include a poor mental health state, lack of self-efficacy, motivation and enjoyment, not valuing or prioritising exercise, and beliefs that exercise may have no positive outcome or even a negative impact on their pain	Angel et al.^4^ Boutevillain et al.^11^ Cook and Hassenkamp^19^ Cooper et al.^21^ Dean et al.^23^ Hay et al.^30^ Joyce et al.^37^ Joyce et al.^36^ Liddle et al.^43^ Mathy et al.^45^ Palazzo et al.^54^ Riipinen et al.^59^ Saner et al.^62^ Slade et al.^67^ Slade et al.^68^ Sokunbi et al.^71^ Stilwell et al.^72^	High confidence	No/very minor concerns regarding methodological limitations, minor concerns regarding coherence, no/very minor concerns regarding adequacy, and no/very minor concerns regarding relevance
Negative social experiences with health professionals, family, and friends that undermine efforts to adhere to exercise. These experiences include poor communication and advice around pain that discourages them and makes them feel unsupported. In addition, a social environment that makes them feel uncomfortable	Boutevillain et al.^11^ Cook and Hassenkamp^19^ Cooper et al.^21^ Joyce et al.^36^ Liddle et al.^43^ Mathy et al.^45^ Palazzo et al.^54^ Slade et al.^67^ Slade et al.^68^ Sokunbi et al.^71^ Stilwell et al.^72^ Yardley et al.^77^	High confidence	No/very minor concerns regarding methodological limitations, minor concerns regarding coherence, minor concerns regarding adequacy, and no/very minor concerns regarding relevance
External factors outside of an individual that hinder the process of organising exercise and worsen the experience of exercise that reinforce exercise nonadherence. These include difficult logistics, lack of accessibility to resources, and exercise environment and type	Angel et al.^4^ Boutevillain et al.^11^ Cook and Hassenkamp^19^ Cooper et al.^20^ Cooper et al.^21^ Dean et al.^23^ Joyce et al.^36^ Mathy et al.^45^ Morris et al.^47^ Palazzo et al.^54^ Saner et al.^62^ Slade et al.^67^ Slade et al.^68^ Sokunbi et al.^71^ Stilwell et al.^72^ Yardley et al.^77^	High confidence	No/very minor concerns regarding methodological limitations, minor concerns regarding coherence, minor concerns regarding adequacy, and no/very minor concerns regarding relevance

## 4. Discussion

This systematic review of qualitative studies generated 4 main themes and 16 subthemes that were barriers and enablers to exercise adherence for people with CLBP. The 4 main themes were (1) exercise, pain, and the body, (2) psychological factors, (3) social factors, and (4) external factors. Within these main themes, subthemes were often both barriers and enablers and predominantly affected adherence before and during exercise. There was moderate to high confidence in the themes generated.

This review identified many barriers and enablers to exercise adherence consistent with a previous earlier review.^69^ Furthermore, our review included additional studies and provided new insights by including studies of people with any exercise experiences and focusing only on the perspectives of individuals with CLBP. This led to the identification of new barriers and enablers including social interactions with family and friends that hindered or facilitated exercise and access to resources (or lack thereof) that affected exercise. These barriers and enablers have been identified in previous studies of pain-free populations,^9,38,51^ and our results suggest they are also relevant to people with CLBP.

One theme identified in this review unique to the musculoskeletal pain literature was “exercise, pain, and the body.” Previous literature^69^ predominantly focused on pain changes, whereas in our review, this theme provided a picture of the variety and complexity of bodily experiences felt by those living with CLBP and how this affected exercise adherence. For example, disability due to back pain (barrier) and regaining function through exercise (enabler) were important factors influencing exercise adherence unique to this population. Furthermore, studies reported that the interplay of pain and exercise was impactful for those experiencing other comorbidities, which is common for those living with CLBP.^33,58^ This finding shows the importance of individualising exercise treatment for people with CLBP to target barriers and enablers unique to their pain experiences and comorbidities, particularly as these were the only 2 barriers and enablers to span the pre-exercise, during-exercise, and post-exercise stages.

A notable result from this review was that 15 of 16 subthemes consisted of both barriers and enablers to exercise adherence. This points towards a reconceptualization of barriers and enablers, showing that they are more appropriately represented on a spectrum rather than being dichotomous. This idea was directly observed in some studies.^11^ For example, a single person's experience of exercise could range from extremely unpleasant to very enjoyable (“after a while you get really bored, it is no longer fun”^11^). However, most studies in this review reported barriers and enablers as separate, binary experiences. This is likely a result of the philosophical underpinnings of qualitative research, where researchers do not aim to provide a representative sample or perspective but instead provide a rich and in-depth slice of the phenomenon under study.^75^ Furthermore, participants are likely to only experience and report specific barriers and enablers to adherence during a clinical trial intervention compared with exercising outside that context. For example, a qualitative study of an exercise trial may include enablers surrounding exercise resources or health professionals, however these may be key barriers in a study of people in a community setting because they may not have access to these services. A strength of this review was that it was able to synthesize a variety of experiences to present a broader analysis across different populations and contexts. This included different settings (hospital, medical centre, physiotherapy clinic, exercise trial vs community-based), health professionals (physiotherapists, chiropractors, and general practitioners), exercise programs (prescribed vs nonprescribed, aerobic, resistance, and flexibility), and people (age, sex, and CLBP duration). Therefore, it is likely that reconceptualizing barriers and enablers as a spectrum of factors accounts for the complexity and nuance that people with CLBP may experience when trying to adhere to exercise in the long term. This finding could be valuable for providing personalised care, as clinicians try to address all relevant factors as well as their level of influence on exercise adherence people with CLBP.

This review used a novel approach to classify barriers and enablers across different time points in relation to exercise, rather than being general to the overall exercise adherence behaviour. This approach fits across various behaviour change models. For example, the theory of planned behaviour^3^ proposes that an individual's attitudes and intentions prebehaviour result in the occurrence of the behaviour, that is, the pre-exercise stage. In addition, the COM-B model^46^ shows how a behaviour or its outcomes (ie, during-exercise and post-exercise stages) can have a positive feedback loop on barriers and enablers leading to reinforcement of that behaviour. Unsurprisingly, barriers and enablers related to the actual experience of exercise, such as enjoyment, environment (social and physical), and exercise type, were identified as most relevant during exercise. Barriers and enablers related to the outcomes of exercise (eg, pain changes, functional improvement, and physiological changes) were identified as relevant after exercise. Most barriers and enablers (11 of 16) were present in the pre-exercise stage, which were defined as the times when people contemplated or planned to exercise. This suggests that the pre-exercise planning stage may be the most critical time point for influencing adherence behaviour. However, this does not completely align with current literature where barriers and enablers from the pre-exercise stage, such as self-efficacy and motivation, have been identified as better predictors of initiation or short-term exercise, rather than maintenance (long-term adherence) of the behaviour.^56^ Interestingly, habit strength and self-identity are better predictors of long-term adherence,^56^ but these barriers and enablers were seldom identified in this review or reported by the previous review.^69^ This may result from some studies in this review extrapolating barriers and enablers to exercise adherence from initial exercise experiences. It is possible that including more studies investigating longer-term exercise programs may have identified these additional relevant predictors of exercise adherence. Therefore, identifying barriers and enablers at their time points of influence in relation to exercise may be a helpful method of addressing certain components of exercise adherence behaviour, but it requires further research to establish its validity. Until further evidence emerges, clinicians should continue to provide personalised care that aims to address all the meaningful barriers and enablers to an individual.

### 4.1. Strengths and limitations

We prospectively registered the study protocol and reported this review according to the ENTREQ guideline. We appraised the methodological quality of the included studies and assessed the certainty of evidence and found that there were few methodological limitations at the study level leading to moderate to high confidence in our themes generated. Furthermore, this review included 23 papers, with 10 new studies compared with the previous review (2 of 15 were excluded from the previous review because of participants not having CLBP). This review also synthesized a wider range of exercise experiences and individuals' perspectives instead of health professionals' views, which contributed to a more comprehensive picture of barriers and enablers to exercise adherence. Furthermore, barriers and enablers were mapped onto the TDF, providing theory-informed domains to approach the task of changing exercise adherence behaviour in people with CLBP. For example, the subtheme motivation was mapped onto the goals domain, which involves providing outcomes/end-states that an individual wants to achieve, that is, providing an avenue to increase motivation.

However, there were also some limitations. We inferred that all patient experiences with exercise interventions (single-session or long-term program) would contribute relevant information on the barriers and enablers associated with exercise adherence. However, this may have led to an overrepresentation of barriers and enablers associated with experiences of single-exercise sessions/short-term programs. Although many of these barriers and enablers are likely to be similar to those associated with adherence to longer-term programs, it is possible that addressing some short-term barriers and enablers may be ineffective for improving long-term adherence. The GRADE-CERQual guidelines do not provide specific methods to judge methodological limitations. Therefore, we relied on our content expertise as exercise physiologists and knowledge of qualitative research to evaluate the confidence in the themes. Barriers and enablers of adherence to other types of physical activity (eg, recreational physical activity such as dance or sport) were not included, which are also likely to be helpful for researchers and medical professionals working with individuals with CLBP. Only studies written in English were included and all studies were conducted in high-income, culturally Western countries. Very limited data were presented on the cultural background, gender identity, education, socioeconomic status, or occupational status of participants. In addition, participants' age or life stage (eg, parenting, retired) were seldom explored as barriers or enablers. Each of these factors could conceivably influence how readily an individual might adhere to exercise, but we cannot comment on this based on the studies included in our review. Finally, few studies (5 of 23, 22%) provided author reflexivity statements, representing a potential source of bias.

### 4.2. Further research

Further research should look to develop a tool/intervention that uses the list of barriers and enablers identified in this review and frames them across a spectrum of experiences specific to their different time points (pre-exercise, during-exercise, and post-exercise). Alternatively, these findings could be used to inform recommendations for researchers and/or clinicians to assess and understand the factors affecting exercise adherence, which could help them to better target their interventions towards the individual's needs, increasing the likelihood of long-term adherence. Further qualitative research should investigate the barriers and enablers across different genders, life stages, and physical activity more broadly as well as culturally and linguistically diverse populations to address barriers and enablers that may be specific to these contexts. In addition, the impacts of habit strength and self-identity as barriers and enablers to exercise adherence should be determined as these are important predictors of long-term exercise adherence.^24,28^

## 5. Conclusion

This systematic review identified and synthesised the available qualitative evidence concerning barriers and enablers to exercise adherence from the perspectives of individuals with CLBP. Many of the identified barriers and enablers were consistent with the previous exercise adherence literature, although some were specific to the experiences of people with CLBP. Our findings suggest that barriers and enablers to exercise adherence may exist on a spectrum and affect adherence at different time points, particularly pre-exercise. Further research is required to develop interventions that can use these findings for a more personalised treatment plan that increases exercise adherence.

## Conflict of interest statement

The authors have no conflicts of interest to declare.

## Appendix A. Supplemental digital content

Supplemental digital content associated with this article can be found online at http://links.lww.com/PAIN/C34.
